# The potential impact of immunization campaign budget re-allocation on global eradication of paediatric infectious diseases

**DOI:** 10.1186/1471-2458-11-739

**Published:** 2011-09-28

**Authors:** Tiffany Fitzpatrick, Chris T Bauch

**Affiliations:** 1 Department of Mathematics and Statistics, University of Guelph, Canada; 2 Department of Epidemiology, Biostatistics, and Occupational Health, McGill University, Canada; 3 Department of Ecology and Evolutionary Biology, Princeton University, USA

## Abstract

**Background:**

The potential benefits of coordinating infectious disease eradication programs that use campaigns such as supplementary immunization activities (SIAs) should not be over-looked. One example of a coordinated approach is an adaptive "sequential strategy": first, all annual SIA budget is dedicated to the eradication of a single infectious disease; once that disease is eradicated, the annual SIA budget is re-focussed on eradicating a second disease, *etc*. Herd immunity suggests that a sequential strategy may eradicate several infectious diseases faster than a non-adaptive "simultaneous strategy" of dividing annual budget equally among eradication programs for those diseases. However, mathematical modeling is required to understand the potential extent of this effect.

**Methods:**

Our objective was to illustrate how budget allocation strategies can interact with the nonlinear nature of disease transmission to determine time to eradication of several infectious diseases under different budget allocation strategies. Using a mathematical transmission model, we analyzed three hypothetical vaccine-preventable infectious diseases in three different countries. A central decision-maker can distribute funding among SIA programs for these three diseases according to either a sequential strategy or a simultaneous strategy. We explored the time to eradication under these two strategies under a range of scenarios.

**Results:**

For a certain range of annual budgets, all three diseases can be eradicated relatively quickly under the sequential strategy, whereas eradication never occurs under the simultaneous strategy. However, moderate changes to total SIA budget, SIA frequency, order of eradication, or funding disruptions can create disproportionately large differences in the time and budget required for eradication under the sequential strategy. We find that the predicted time to eradication can be very sensitive to small differences in the rate of case importation between the countries. We also find that the time to eradication of all three diseases is not necessarily lowest when the least transmissible disease is targeted first.

**Conclusions:**

Relatively modest differences in budget allocation strategies in the near-term can result in surprisingly large long-term differences in time required to eradicate, as a result of the amplifying effects of herd immunity and the nonlinearities of disease transmission. More sophisticated versions of such models may be useful to large international donors or other organizations as a planning or portfolio optimization tool, where choices must be made regarding how much funding to dedicate to different infectious disease eradication efforts.

## Background

Infectious diseases have long imposed a considerable burden on human populations around the world [1-3]. However, the only vaccine-preventable infection to have been globally eradicated to date is smallpox. Although the smallpox vaccine was available starting in the 19^th ^century, smallpox was only eradicated in 1977 after a final, intensive 10-year push by the World Health Organization that required global coordination of control efforts [3].

Vaccine-preventable paediatric infectious diseases such as polio, measles and pertussis continue to cause significant morbidity and mortality worldwide. While polio has been successfully eradicated from the western hemisphere, it remains endemic in four countries (Nigeria, Afghanistan, India, and Pakistan) [4]. An estimated 1 in 200 polio infections lead to irreversible paralysis and 5-10% of these individuals will die due to breathing complications resulting from this paralysis [5,6]. Deformities and paralysis acquired as a result of polio infection have lasting lifelong implications. Initial symptoms include fever, headache, fatigue and vomiting [7]. Some of these initial symptoms are similar to those of common ailments such as influenza, making infection control difficult. Measles likewise is responsible for considerable morbidity and mortality. Most measles-related deaths are due to complications such as dehydration as a result of diarrhea, or pneumonia [7-10]. Pertussis (whooping cough) infection is most serious in infants under 6 months of age: 1 in 200 of these infants will die as a result of complications, such as brain damage [6,10]. Pertussis can be contagious up to three weeks after infection, and it is most contagious during the first two weeks [11,12]. Symptoms at this stage closely resemble those of a common cold, meaning a person may not notice they are infected with pertussis, and can unknowingly transmit the pathogen [12].

Immunization for many paediatric infectious diseases in lower income countries is delivered not only through routine immunizations, as in higher income countries, but also through large-scale campaigns known as supplementary immunization activities (SIAs) [4,5,13]. SIAs improve health outcomes in low-income countries by preventing the build-up of the susceptible population and thereby preventing outbreaks [14,15]. At the time of submission of this paper, polio is close to eradication and remains endemic in only four countries (India, Nigeria, Afghanistan, and Pakistan), thanks largely to SIAs [4]. However, as long as a transmissible disease remains endemic in any one country, it can resurface in other countries through case importation.

SIAs also provide a platform for delivering other interventions such as Vitamin A supplements; insecticide treated bednets (ITNs) for the prevention of malaria; and anti-viral drugs. A recent economic study concludes that integrating ITN distribution for malaria prevention into measles vaccination campaigns can achieve greater and more equitable coverage of ITN, while also reducing the total cost of bednet procurement from US $3.42 per bednet distributed to US $0.32 per bednet distributed [14]. This illustrates the potential synergies of coordinating infection control programs for different infectious diseases.

Vaccinating every single person in the world is implausible, and hence eradication of an infectious disease requires the indirect protection offered to unvaccinated individuals through herd immunity. Disease transmission models can capture herd immunity effects and thus be used to project the time to eradication under different immunization scenarios. More generally, they can be used to project a broad range of future health or economic outcomes under different possible intervention strategies.

A number of transmission models of routine and campaign immunization in low-income settings have been developed [16-22]. Most of these focus on a single disease and a single intervention in a single region or country. For instance, a mathematical model has been used to explore the possible implications of various HIV control strategies, such as spreading donor money evenly over a 20-year period versus spending the same amount of money up-front over a 5-year period in an effort to eradicate HIV [16]. The authors' model suggests that spending all funds up-front over a 5-year period could eradicate HIV and result in significant cost-savings, whereas under the same conditions HIV could not be eradicated under the 20-year strategy. The model illustrates how budget allocation strategies can interact with the inherent nonlinearities of disease transmission (and in particular, herd immunity effects) to determine outcomes.

Examples of transmission models for studying the impact of coordinated efforts to control multiple infectious diseases are more rare. However, a model has been used to analyze priority shifting in the funding of programs for two hypothetical vaccine-preventable infectious diseases [17]. The authors concluded that a re-allocation strategy based on a goal of eradication (where all funds are focused to one disease until it is eradicated, at which points funds are re-allocated to focus on the remaining disease) can often achieve better long-term outcomes than a strategy that allocates resources based on which disease has the highest current prevalence ("fire-fighting") or a simultaneous strategy that divides resources equally among the control programs.

Here, we explore such re-allocation strategies using a transmission model for three hypothetical infectious diseases (A, B and C) against which SIAs are conducted in each of three low-income countries that have not yet eliminated the diseases. We use a transmission model to project the time required to eradicate each of these diseases under various possible SIA budget allocation strategies. For each country, a total budget is allocated for use in SIAs for all three infectious diseases. We assume it is possible for a central decision-maker to allocate different amounts of money each year among SIA programs for the three diseases. For instance, a decision-maker can adopt a "sequential strategy" of initially allocating all SIA budget to Disease A until it is eradicated (meaning no budget is available for SIAs against Diseases B and C during this period, but routine coverage for B and C continues), and then once Disease A is eradicated, the annual SIA budget can be dedicated to Disease B until it is eradicated, and then finally to Disease C. Herd immunity suggests that all three diseases may be eradicated more quickly under this approach than under approaches that do not strategically re-allocate among the SIA programs. Alternatively, a decision-maker can opt for a "simultaneous strategy" of simultaneously funding SIAs for all diseases.

Our analysis differs from that of Ref. [17] in that it explores three diseases in three populations instead of two diseases in one population; it includes case importation; it systematically explores how model predictions depend on input parameter values; it explores scenarios such as (1) constant versus increasing routine immunization coverage, and (2) what happens when SIA budget for all three diseases is interrupted for a period of time such as due to lack of donor funds. Our results generally confirm the previous finding that sequential strategies can work better than simultaneous strategies for certain budget levels. However, we also find that the relative effectiveness of the sequential strategy can depend sensitively on details such as how much case importation there is between the three countries, how much budget is available, and the order in which the diseases are eradicated.

Our model structure and assumptions are deliberately simplified, because our goal is to illustrate how budget allocation strategies can interact with the nonlinear nature of disease transmission and herd immunity effects to drive surprisingly large and disproportionate differences in the time required to eradicate infectious diseases under different budget allocation strategies. A fuller evaluation of this type for use in formulating policy would require greater attention to how transmission is modelled and how costs and health outcomes are estimated and compared. It would also require incorporating the restrictions and realities of how international donors support disease control programs, and a fuller accounting for the disruption to health services delivery caused by moving budget between programs. Notwithstanding, the herd immunity effects captured by our simple model are shared by more complex infectious disease transmission models, therefore a more sophisticated analysis might share some of the same qualitative features we will highlight here.

## Methods

### Mathematical Model

Our model structure and parameter values are intended to represent three hypothetical paediatric infectious diseases. More sophisticated country-specific and disease-specific versions of such transmission models would be required to address the issue of optimal eradication strategies for actual infectious diseases

We use a compartmental transmission model. This type of model has been shown to agree reasonably well with time series of infection incidence many paediatric infectious diseases even when age structure is not included [12,20,23,24]. Individuals can be Susceptible, Infectious, Recovered, or Vaccinated with immunity, hence we refer to it as a SIRV model. The model assumes long-term natural immunity, which is a reasonable approximation for many paediatric infections [12,24]. The model equations for a given disease in country *k *are given by

(1) $$\begin{array}{c} \frac{dS_{k}}{dt}=\mu \left(1-\varphi \varepsilon \right)N_{k}-\beta S_{k}\frac{I_{k}}{N_{k}}-\mu S_{k}\\ \frac{dI_{k}}{dt}=\beta S_{k}\frac{I_{k}}{N_{k}}-\gamma I_{k}+mI_{a}+mI_{b}-2mI_{k}-\mu I_{k}\\ \frac{dR_{k}}{dt}=\gamma I_{k}-\mu R_{k}\\ \frac{dV_{k}}{dt}=\mu \varphi \varepsilon N_{k}-\mu V_{k} \end{array}$$

where all model parameters and associated baseline values are defined in Table 1 [7,8,12,25-28]. These equations represent the change over time of the number of individuals who are susceptible (*S*_k_), infectious (*I*_k_), recovered (*R*_k_) and vaccinated (*V*_k_) in country *k*. The parameters are generally country-specific and disease-specific (although subscripts are omitted for clarity). Parameters include the per capita birth rate (μ), the per capita all-causes death rate (also μ), the rate at which individuals recover from infection (γ), and the efficacy of vaccination (ε). Vaccine was assumed to have "all or nothing" efficacy. The *βSI/N *term describes the transmission of infection from infected to susceptible persons and means that the rate at which new infections are created is proportional to the product of the number of infected and susceptible individuals. The *mI*_a _and *mI*_b _terms represent case imports to country *k *from the other two countries *a *and *b*, where *m *is the per capita import rate. Conversely, the -2 *mI*_k _term represents cases leaving country *k *and being exported to countries *a *and *b*. For simplicity, we have assumed the *per capita *importation rates to be the same between countries even though they will generally vary, and will be higher for countries in close geographic proximity. The transmission rate, *β*, was computed from estimates of the recovery rate, γ, and the basic reproductive number, *R_O_*, from the relation *β*≈*γR_O _*for the three diseases [12]. We defined an infection as eliminated in a country if the number of infectious persons dropped below one, in which instance the number of infectious persons was set to zero. This was done to capture some of the effects of stochastic extinction; a complete accounting for such stochastic effects would require a stochastic model. We explore the effects of changing this elimination threshold in sensitivity analysis. If the infection is eliminated in all three countries, it is considered eradicated. We did not include a 3-year waiting period before elimination is certified in a given country and resources are switched to a different immunization program, although this should not significantly change the relative success of the sequential versus simultaneous strategies. This model neglects disease-related deaths, however, this will not change the qualitative feature of the dynamics or the time to eradication since death often occurs after the period of peak infectiousness for many paediatric infectious diseases.

**Table 1:** Model parameters

Parameter	Definition	Value(s)	References
Disease-specific parameters			

*φ*	Routine vaccination rate	0.5/year	assumed

*γ*	Recovery rate	Disease A: 1/19 per day* Disease B: 1/14 per day* Disease C: 1/30 per day*	[12]

R_O_	Basic reproductive number	Disease A: 6* Disease B:15* Disease C: 14*	[12]

*ε*	Efficacy of vaccine after 1 dose	Disease A: 99%* Disease B: 95%* Disease C: 90%*	[7,8,25]

Country-specific parameters			

*m*	Case importation rate	0.0001 per year per capita in all countries	assumed

*μ*	Birth/death rate (per 1, 000 per year) [As of 2009]	India: 21.72/1000/yr Nigeria: 36.65/1000/yr Afghanistan: 38.37/1000/yr	[25-28]

*N*	Total population [As of 2009]	India: 1, 156, 897, 766 Nigeria: 149, 229, 090 Afghanistan: 28, 395, 716	[25-28]

*The parameter values for diseases A, B and C are hypothetical but plausible, being based on values for common paediatric infections such as measles, pertussis, and polio. (However we note that *R*_0 _can vary between populations and over time, and efficacy for oral polio vaccines can be as low as 50%).

We assume that routine vaccination is administered in the first year of life. The fraction of the population who are successfully vaccinated under routine vaccination is defined by the *φεN *term in the equations, where *φ *is the rate at which individuals are vaccinated and *N *is the population size, which together with *μ *determines the number of births and hence the number of individuals vaccinated. Immunization campaigns (SIAs) are modelled by moving individuals from the susceptible to the vaccinated compartment at the time of the campaign, according to assumed SIA coverage and vaccine efficacy. We assumed that SIAs could be conducted rapidly in a given country, since they are usually conducted as large-scale campaigns. Hence, SIAs were modelled as a "pulse" to the differential equations, resulting in the following instantaneous changes to the compartment sizes:

$$\begin{array}{c} S_{k}\to S_{k}-S_{k}\varphi_{SIA}\varepsilon \\ V_{k}\to V_{k}+S_{k}\varphi_{SIA}\varepsilon \end{array}$$

where *φ*_SIA _is the vaccine coverage in the SIA. Vaccine coverage and efficacy vary according to infectious disease and SIA program (i.e. the vaccine for some diseases will intrinsically have a higher efficacy than others). We note that real-world SIAs involve choices about which age groups to immunize, but our model is not age-structured, hence the implicit assumption that vaccine is administered regardless of age.

### Assumptions about study diseases and populations

Our three hypothetical diseases were Disease A (low transmissibility), Disease B (higher transmissibility, shorter infectious period), and Disease C (higher transmissibility, longer infectious period). The basic reproductive numbers *R*_0 _for Diseases A, B, and C were taken to be 6, 15, and 14, respectively [12]. We used India, Nigeria and Afghanistan as our three case study countries, corresponding respectively to one country each of large, moderate and small population size in relative terms.

### Vaccine coverage and SIA budget scenarios

We designed simplified scenarios for the implementation of immunization programs. We introduced routine vaccination for all three diseases in all three countries in 1960. In 1990, we introduced SIAs in addition to routine vaccination; a total annual budget was allocated for SIAs for all three diseases in all three countries. Each country was allocated their own SIA budget in direct proportion to their population size, and each country divided this allocated budget equally among SIAs for the three infectious diseases. (We note that in practice, the assumption that budget for SIAs is divided equally across the three diseases may not hold. However, the actual criteria for how budget is allocated across SIAs may be numerous and complex, hence we opted for this simplifying assumption.) The budget for each SIA program in each country was translated into SIA coverage by dividing the budget by the unit vaccine costs in Table 2 to obtain number of doses administered per SIA. We assumed that both susceptible and naturally immune individuals were vaccinated during SIAs. The vaccine unit costs include the cost of the vaccine and cost of vaccine administration. Vaccine costs varied from country to country, and are dependent on which vaccine is being given. Table 2 shows the assumed prices of each vaccine in India, Nigeria and Afghanistan, which were estimated or taken directly from the literature [10,13,14,29-32]. SIAs were assumed to occur every 3 years in the baseline scenario, and budget money from years where no SIA was conducted could be saved and used for SIAs in subsequent years. Hence, under certain values of the annual budget, the SIA coverage might vary depending on whether SIAs were conducted every 2, 3 or 4 years. We note that assumed budgets in the model reflect only the budgets for SIA programs, and not for routine immunization. We also note that prices can vary according not only to vaccine type but also country and type of vaccine delivery program, and that Table 2 required making assumptions about unit costs when good country-specific data were not available.

**Table 2:** Vaccination price for each country by disease

Price of Vaccination ($US)		References
Disease A*	India: 0.31 Nigeria: 1.15 Afghanistan: 0.31	[13,29,30]

Disease B*	India: 0.64 Nigeria: 0.76 Afghanistan: 0.78	[14,10,31]

Disease C*	India: 0.62 Nigeria: 0.62** Afghanistan: 0.62**	[32]

* The parameter values for diseases A, B and C are hypothetical but plausible, being based on values for common paediatric vaccines such as monovalent measles vaccine, dTaP and oral polio vaccine.

** Assumed value due to lack of data.

Starting in 2010, we distinguished two possible strategies. Under the "simultaneous strategy", funding continues to be divided equally among the three diseases in the same way as it was done between 1990 and 2009 (i.e. efforts to eradicate the infectious diseases continue to occur concurrently). In contrast, under the "sequential strategy", budget reallocation starts in 2010 such that all SIA funding in each country is dedicated to SIAs for one disease until that disease is eradicated; once the first disease is eradicated, then beginning in the next round of SIAs, all SIA funding for the country is then dedicated to the next disease until that is eradicated; once the second disease is eradicated then all funding is dedicated to the last disease commencing in the subsequent round of SIAs until it is eradicated. All countries adopt the same order of eradication, and all countries maintain a routine vaccination rate of 50% for all three diseases during this time. Given that all funding goes into eradication of one disease at a time, at any given time at least two diseases are receiving no funding, meaning no SIAs would occur for them during this period. In the baseline scenario we considered eradication in the order Disease A, B, then C; other orders of eradication were also analyzed.

### Simulations

It is possible to solve the equilibrium of the SIRV equations analytically, resulting in a simple expression for the vaccine coverage required to eliminate an infection in a population. When there is enough budget to exceed this threshold for all three diseases in all three populations, then all three diseases could eventually be eradicated with a simultaneous strategy [17]. If less budget is available, a sequential strategy might work better, and in this case the SIRV equations need to be simulated to determine how much time is required to eradicate the infection under simultaneous versus sequential strategies.

We simulated the model using MATLAB R2008a. There were 9 sets of equations in total, of the same structure as equation system (1): three for each disease in each of the three countries. The initial conditions were such that the number of susceptible, infected, and vaccinated persons for a single disease in a given country were equal to 5%, 0.1%, and 0% of the country's total population, respectively (the remainder were recovered). However, the simulation was run for a sufficient amount of time for the prevalence of each infection in each country to come to equilibrium by the year 1960 when routine immunization begins. The routine vaccination rate was 0.5 per year, each year, in the baseline scenario, although we explored the case where it increases at 0.01 per year from an initial level of 0.5 per year from 2010 onward in a sensitivity analysis.

## Results

**Figure 1:**
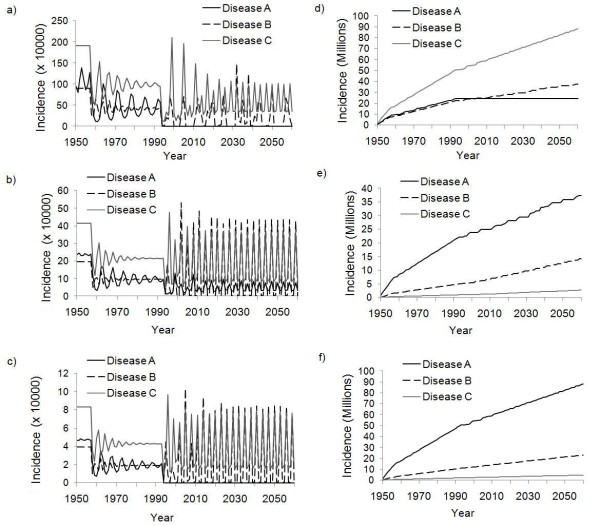
**Time series of prevalence under simultaneous strategy**. Disease prevalence in India (a), Nigeria (b), and Afghanistan (c) according to simulations, where routine vaccination is introduced in 1960 and SIAs are introduced in 1990. Cumulative incidence for India (d), Nigeria (e), and Afghanistan (f) is also shown for the same scenario.

The impact of changes in vaccine coverage in 1960 (introduction of routine vaccination) and 1990 (introduction of SIAs) can be seen in time series of disease prevalence of all three diseases in all three countries under the simultaneous strategy (Figure 1). Baseline parameter values (Table 1) are used for this simulation as well as subsequent simulations, except where otherwise noted. In 1960, the introduction of routine vaccination induces transient oscillations in infection prevalence until the model dynamics "settle down" to a new equilibrium prevalence that is lower than the pre-vaccine era prevalence in all three countries. In 1990, the introduction of SIAs with a total annual budget of US $180 million for all three SIA programs in all three countries results in further reduction in prevalence for all three infections: Disease B and Disease C remain endemic in all three countries, and Disease A is eliminated from India and Afghanistan although it remains endemic at very low levels in Nigeria. Hence, under the simultaneous strategy for baseline parameter values, no disease is eradicated for the foreseeable future. Therefore, a total annual budget of US $180 million is used as our baseline parameter value, to provide a meaningful contrast to outcomes under the sequential strategy versus the simultaneous strategy. This value is on the same order of magnitude as immunization program budgets in some countries [16,21,29,30].

The differences in epidemic patterns between the three countries observed in Figure 1 are due to differing birth rates (Table 1) and vaccine unit costs (Table 2). The time between outbreaks is longer in India (Figure 1a) than in Nigeria (Figure 1b) and Afghanistan (Figure 1c) because India has a lower birth rate than either Nigeria or Afghanistan (Table 1). Similarly, Disease A persists for longer in Nigeria (Figure 1b) than in India (Figure 1a) or Afghanistan (Figure 1c) because the vaccine for Disease A costs significantly more for Nigeria than for India or Afghanistan (Table 2). The way that elimination is defined may also be relevant: we considered a disease as eliminated in a country if prevalence fell below one person, and this benchmark is harder to reach in a larger population such as that of India.

**Figure 2:**
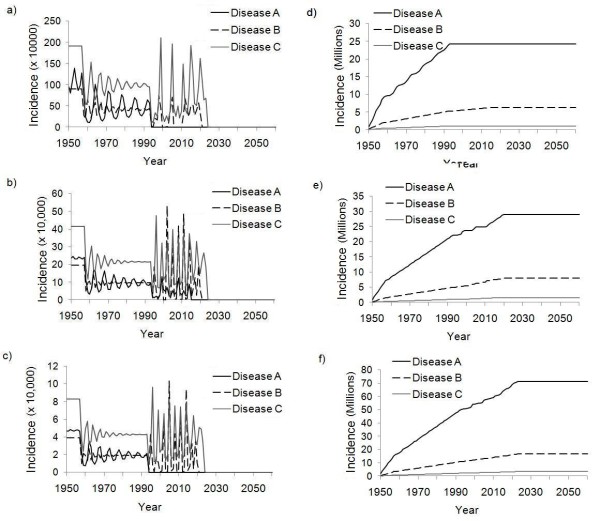
**Time series of prevalence under sequential strategy**. Predicted disease prevalence in India (a), Nigeria (b), and Afghanistan (c), such that routine vaccination is introduced in 1960, SIAs begin in 1990, and reallocation occurs according to the sequential strategy in 2010, eradicating diseases in the order: A, B, then C. Cumulative incidence for India (d), Nigeria (e), and Afghanistan (f) is also shown for the same scenario.

**Figure 3:**
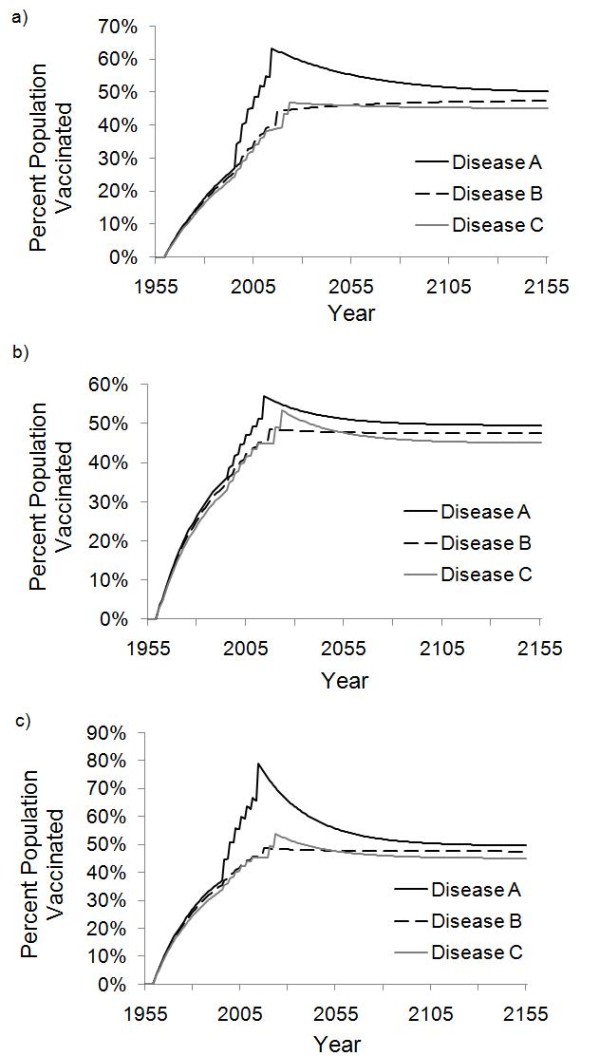
**Time series of vaccination coverage under sequential strategy**. SIA vaccination coverage in India (a), Nigeria (b) and Afghanistan (c) under the baseline sequential with an annual SIA budget of $180 million.

In the case of the sequential strategy with the same annual budget of US $180 million, the time evolution is the same as for the simultaneous strategy until budget reallocation begins in 2010. Under the sequential strategy, the model predicts a very different time evolution of future incidence (Figure 2). All three diseases are eradicated by 2030: Disease A is eradicated first in 2021, followed by Disease B in 2024, and finally Disease C in 2030 (by comparison, under the simultaneous strategy for the same budget, no disease is eradicated in the foreseeable future). Figure 3 shows a time series of SIA vaccination coverage in each country under the sequential strategy, and the changing vaccine coverage due to budget re-allocation is apparent.

This example demonstrates how budget reallocation strategies can take advantage of herd immunity to speed eradication of all three diseases. However, there are tradeoffs: in the period where SIA funds are being dedicated entirely to Disease A, the average prevalence of Diseases B and C increases for a period after budget reallocation begins in 2010. This is seen in Figure 2 versus Figure 1 in the decade after 2010, where recurrent epidemic spikes are present in both figures but the average prevalence of Diseases B and C is higher in Figure 2 than in Figure 1. This is also seen in plots of cumulative incidence over time, which also appear in Figures 1 and 2.

We considered a disease to be eliminated in a country once prevalence in that country fell below 1 person. To test the impact of this assumption, simulations were also conducted using elimination thresholds of 2, 0.1 and 0.01 persons for the baseline sequential strategy. We found that the year of eradication of all three diseases changed from 2030 (for the baseline scenario where the threshold is 1 person) to 2024, 2060 and 2066 under these two thresholds, respectively. Hence, the cutoff for defining elimination in a country has some impact on model predictions although the differences are not large enough to change our conclusion that the sequential strategy outperforms the simultaneous strategy, at least for this level of annual budget.

In the following subsections we explore a wider range of possibilities, including what happens under: (1) variation in the total annual SIA budget; (2) variation in the frequency of SIAs; (3) variation in the order of eradication; (4) interruption in SIA budgets; (5) increasing routine vaccination over time; and (6) varying case importation rates.

### Impact of varying total budget

**Figure 4:**
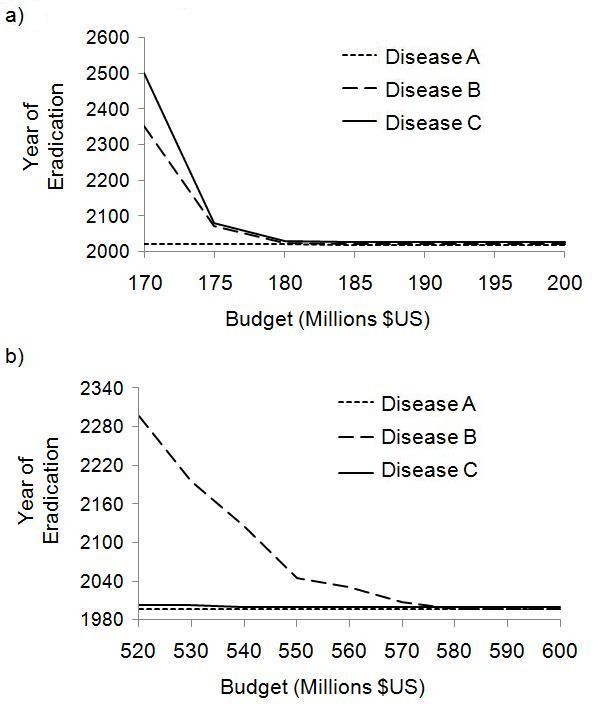
**Year of eradication as a function of annual budget, 3-year SIA intervals**. Year of eradication as a function of annual SIA budget under a 3-year SIA interval, for the sequential strategy (a) and the simultaneous strategy (b).

We varied the annual SIA budget to understand the impact of budget on time to eradication under the simultaneous strategy versus the sequential strategy. For the sequential strategy, there is a minimum annual budget of approximately $170 million below which eradication of all three diseases never occurs. Above this threshold, the time required to eradicate all three diseases drops sharply. Beyond an annual budget of $190 million, the time to eradication stabilizes and does not decrease significantly as annual budget increases (Figure 4a).

We define an annual budget to be optimal if it requires the least total expenditure, i.e. the sum of annual budgets from the time SIAs are implemented (2010) to the time of eradication is minimized. For the sequential strategy, the optimal annual budget is US $185 million, under which eradication of Diseases A, B and C occurs by 2018, 2021, and 2027, respectively for a total of $3.14 billion between 2010 and 2027. For larger annual budgets, the time to eradication remains the same, such that eradication of all three diseases can be achieved by 2027 under annual budgets of $190, $195, and $200 million. However, the total expenditure required for each of these annual SIA budgets is higher at $3.23, $3.32, and $3.40 billion respectively, between 2010 and 2027.

In summary, under the sequential strategy there is a minimum threshold annual budget required for eradicating all three diseases, and there is also slightly larger optimal annual budget where all three diseases are eradicated for the least aggregate cost over time. Beyond the optimal annual budget, the incremental gains of increased annual budget on time to eradication are small, under our model assumptions.

The impact of annual SIA budget on time to eradication is qualitatively similar under the simultaneous strategy, with both a minimum threshold annual budget and an optimal annual budget. However, the time to eradication is considerably longer for the same or higher annual budget (Figure 4b). For instance, under the minimal threshold annual budget of $520 million, eradication of Diseases A and B occurs by 1997 and 2003 respectively, but Disease C is not eradicated until 2297. For a higher annual budget of $560 million, eradication of Diseases A, B and C occurs by 1997, 2000 and 2031, respectively (Figure 4b)-hence, the time to eradication under the simultaneous strategy is similar to the time to eradication under the sequential strategy but it requires a much higher annual budget ($560 versus $180 million). If the annual budget under the simultaneous strategy is increased further to $580 million, we obtain the (non-historical) result that all three diseases are eradicated by 1997, requiring a total of $4.1 billion from 1990 to 1997. This total value of $4.1 billion from 1990 to 1997 under the optimal simultaneous strategy is actually less than the total value of $6.8 billion from 1990 to 2027 (year of eradication) under the optimal sequential strategy. Hence, a simultaneous strategy could eradicate all three diseases more quickly and for less money expended overall than a sequential strategy, but the annual budget must be three times higher than usual ($580 million versus $180 million), which may be difficult to achieve.

### Impact of varying SIA scheduling

**Figure 5:**
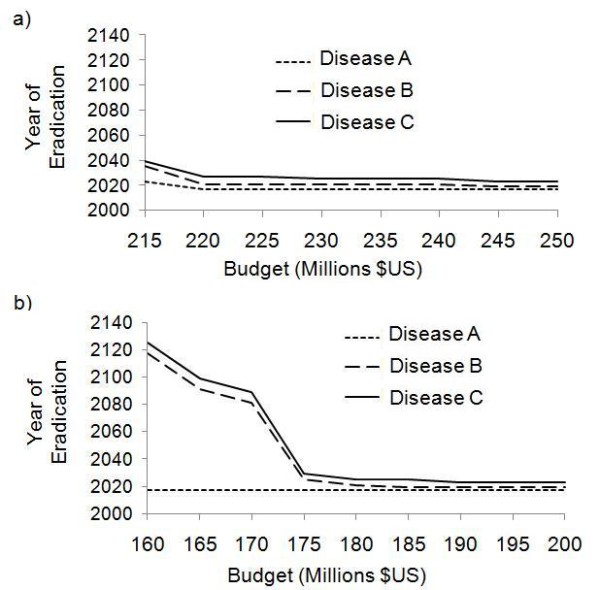
**Year of eradication as a function of annual budget under 2-year and 4-year SIA intervals**. Year of eradication as a function of annual SIA budget for the sequential strategy if SIAs occur either every 2 years (a) or every 4 years (b).

We next analyze the impact of time interval between SIAs on the time and budget required to eradicate all three diseases, under the sequential strategy. If SIAs occur every two years instead of every three years, there is still a minimum threshold budget ($220 million) below which eradication never occurs. Above this threshold there is very little variation in time to eradication, as occurred when SIAs were held every three years. For the optimal budget of $220 million, eradication of Diseases A, B and C, occur in 2017, 2021, and 2027 respectively (Figure 5a). Utilizing this optimal budget, a 2-year SIA schedule would require a total funding of $3.74 billion between 2010 and 2027.

If SIAs occur every 4 years, the minimum threshold budget is $160 million. Above this threshold, there is a sharp decline in the number of years required for eradication, but beyond an optimal budget of $180 million, the time to eradication does not significantly decrease. This was also a similar pattern as observed with the 3-year schedule. At the optimal budget of $180 million, we see that the eradication of Diseases A, B and C occurs by 2017, 2021, and 2025, respectively, requiring a total of $2.70 billion.

The optimal annual budgets are $3.74, $3.14, and $2.70 billion for the 2, 3, and 4-year SIA schedules respectively; optimal annual budgets are higher for more frequent SIAs. This occurs because annual budgets are fixed and hence SIA coverage is a function of the interval between SIAs; for the same budget, SIA vaccine coverage is higher when SIAs are less frequent. A higher SIA coverage is more likely to push prevalence below the eradication threshold just after the SIA is completed, and if SIAs are coordinated and occur simultaneously in the three countries, then it is more likely that the disease will be eliminated in all three countries. In contrast, for a shorter SIA interval (e.g. every 2 years), fewer individuals are covered during a given SIA and so it is less likely that incidence will fall below the eradication threshold immediately following the SIA. This effect may not occur if SIAs are un-coordinated and occur in different years in each country, which is the actual practice. The effect of SIA interval is likely also a function of how natural immunity develops in the intervals between SIAs. Finally, these differences apply at the optimal annual budget: for higher annual budgets such that very high coverage can be achieved even when SIAs are held every two years, we expect the time to eradication to be shorter for more frequent SIAs.

### Order of eradication

Our baseline sequential strategy eradicates in the order Disease A-B-C, partly on the grounds that Disease A has the lowest basic reproductive number, *R*_0 _= 6, meaning that lower vaccine coverage would be required to eradicate it. Hence, Disease A might represent the "low hanging fruit" to policy makers. Here we explored five alternative orders to understand the implications of ordering for time to eradication: (1) A-C-B, (2) B-C-A, (3) B-A-C, (4) C-A-B, and (5) C-B-A.

We found that the minimum threshold budget remains $180 million for each of the five alternative orderings. The year of eradication for the three diseases under all six possible orderings under a budget of $180 million is shown in Table 3. From this table it is clear that Disease A is eradicated fastest under the four following orderings: A-B-C, A-C-B, B-A-C, and C-A-B; that is, any order attempting to eradicate Disease A either first or second in the series. Disease B is eradicated fastest under either of the two orders where funding is first allocated towards its eradication (B-A-C and B-C-A). Under these two orders, eradication of Disease B can occur in 2015. Similarly, Disease C is eradicated fastest under the orderings beginning with Disease C: C-A-B and C-B-A. Interestingly, eradication of all three diseases occurs fastest (by 2027) for the order B-C-A or B-A-C, i.e., for strategies where Disease B is eradicated first (not Disease A which has the lowest *R*_0_). Eradication of all three diseases takes longest (by 2186) for the order A-C-B and also takes very long (until 2177) for the order C-A-B, i.e.--these are strategies where Disease B is eradicated last.

**Table 3:** Time to eradication for alternative orderings under an annual SIA budget of $180 million.

Re-allocation order	Year of Eradication		
	
	Disease A	Disease B	Disease C
A - B - C	2021	2024	**2030**

A - C - B	2021	**2186**	2027

B - C - A	**2027**	2018	2024

B - A - C	2021	2018	**2027**

C - A - B	2021	**2177**	2018

C - B - A	**2075**	2072	2021

Bold type indicates year that last disease is eradicated under a given reallocation order.

**Figure 6:**
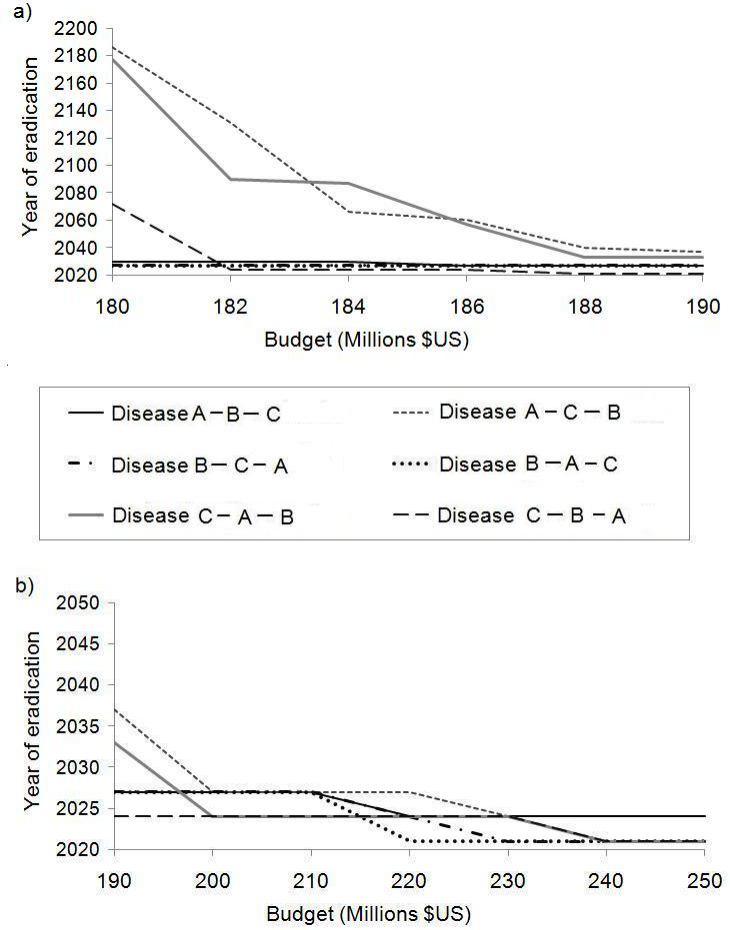
**Change in order of eradication**. Year of eradication under different reallocation orders (A-B-C; A-C-B; B-C-A; B-A-C; C-A-B; and C-B-A) for the sequential strategy with SIA budgets between $180 and $190 million (a) and budgets greater than $190 million (b).

For budgets between $180 and $190 million, the time to eradication increases a great deal if Disease C is eliminated before Disease B (Figure 6a). Beyond $190 million, the time to eradication is roughly the same under all six orderings, such that eradication of all three diseases is possible by 2027 (Figure 6b). The large difference in time to eradication for budgets between $180 and $190 million for different orderings may be due to the high transmissibility of Disease B, which requires higher coverage to eradicate than Disease A or C. Similarly, any order that aims to eradicate Disease B last requires the greatest amount of total funding, even under their respective optimal annual SIA budgets.

It is important to note that while both Disease B and C are highly transmissible, the basic reproductive number of Disease B is slightly higher than that of Disease C (15 and 14, respectively); Disease A has the lowest basic reproductive number, 6. Thereby, we propose that transmissibility accounts for the difference in time to eradication seen under each order: eradication occurs more quickly if the disease with highest transmissibility is eradicated first; eradication takes longest in the reverse scenario, where the disease with the lowest transmissibility is first to eradicated. We have not explored the impact of natural history and transmissibility assumptions systematically, hence other conclusions may emerge if these factors are explored more exhaustively.

### Discontinuity in SIA Funding

We examined any possible consequence of an interruption in SIA funding for the success of the sequential strategy. We examined three different durations of interruption-3, 6, and 9 years-each beginning in 2016. During these periods, SIA coverage was 0% for all three diseases in all three countries.

**Figure 7:**
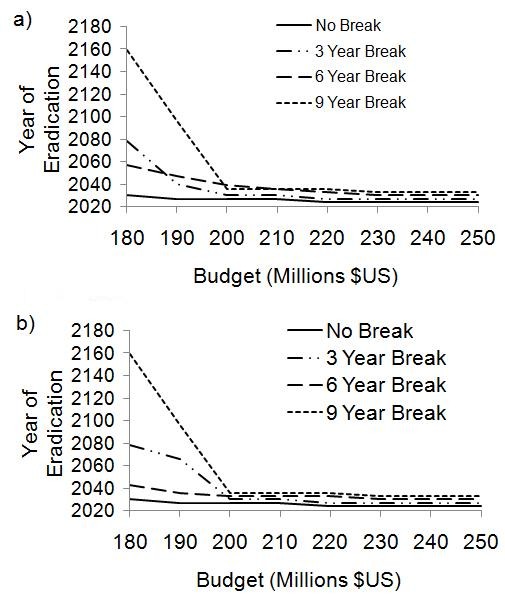
**SIA funding hiatus**. Effect of a break in SIA funding on year of eradication under different annual budget scenarios. Funding is entirely cut (SIA vaccination coverage is 0%) for 0, 3, 6 and 9 years in either all three countries (a) or Nigeria only (b). In each case, the hiatus begins in 2016, and funding re-commences as normal after the hiatus.

As in previous simulations, there is a minimum threshold budget, below which eradication is not possible within the near future. As for the sequential strategy without budget interruption, the minimum threshold budget is $180 million and there is a sharp decline in the number of years required for eradication above this threshold. Beyond a budget of $200 million, increases in budget do not translate into significant differences in time to eradication. In these respects, the results under budget interruption are qualitatively similar to the results under no interruption. However, there are quantitative differences: the time to eradication generally increases under the budget interruption scenario. For budgets greater than the optimal budget of $200 million, interruptions of *N *years translate to delayed eradication by approximately *N *years: the baseline case with no interruption achieves eradication by 2024 under a $220 million budget; the case with a 3-year break requires 3 extra years for the same $220 million budget, the case with a 6-year break requires 6 more years for a $230 million budget, and the case with a 9-year break requires 9 more years for eradication for a $230 million budget (Figure 7a). In stark contrast, below the optimal budget of $200 million, the time to eradication increases enormously for relatively small interruptions (Figure 7a). For example, a 3-year break at a budget of $180 million delays the time to eradication of all three diseases from 2030 to 2080, and the setbacks are even greater for 6-year and 9-year breaks.

Short-term interruptions in annual SIA budgets can also have large implications for total required expenditures from 2010 to eradication. For instance, under a $180 million budget, a 3-year interruption would require a total of $11.70 billion over 68 years (2010-2078) to eradicate all three diseases. Similarly, a total of $7.38 billion is required to eradicate over 47 years under a 6-year interruption in SIA funding, and a total of $25.20 billion is required over 149 years under a 9-year interruption. Without any interruption in funding, it only takes $3.6 billion over 20 years (2010-2030) for the same annual budget of $180 million.

A similar pattern is seen when funding is interrupted in just one country. For instance, in the case of interruptions only in Nigeria when the usual annual budget is $230 million, a 3-year interruption again requires 3 extra years, a 6-year interruption again requires 6 more years, and a 9-year interruption again requires 9 more years (Figure 7b). This occurs because continued endemic infection in Nigeria can act to seed case imports in other countries, necessitating continued control efforts in those countries. For annual SIA budgets below $200 million, a small interruption translates into very long delays in the time to eradication of all three infections in all three countries, as was observed in Figure 7a for the case of interruptions in all three countries. Hence, budget interruptions in SIA funding for any one country can have significant implications for time to global eradication, especially when budgets are close to the optimal or minimal thresholds. These results echo experiences with polio elimination in Nigeria, where there was a cessation of polio immunization between 2002 and 2003 due to a vaccine "scare". As a result, polio spread to other countries and polio eradication was detrimentally affected in the whole region [33].

### Increasing routine coverage over time

For the results reported thus far in this paper, we assumed routine vaccination began in 1960 for each of the three diseases and remained constant at 50% in all three countries. However, vaccine coverage has been increasing steadily over the past few decades and are high for most vaccines in most countries, with a few exceptions such as Nigeria, India and Afghanistan [23]. To understand the impact of our baseline assumption of constant vaccine coverage, we repeated the analysis presented in Figure 4, except that in 2010 routine vaccine coverage begins to increase additively by 1% per year in all three countries until coverage reaches 95% in 2055, after which it remains at 95%.

**Figure 8:**
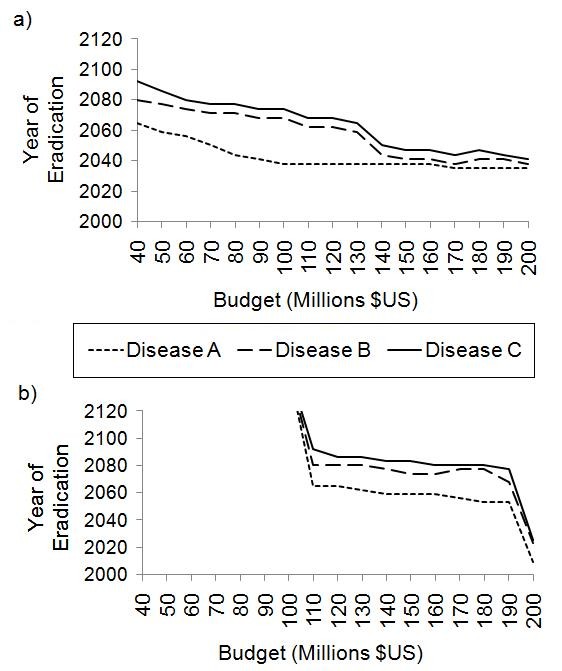
**Increasing routine vaccination coverage**. Year of eradication for a range of annual SIA budgets while routine coverage increases by 1% each year starting in 2010, until it reaches 95%, for the sequential strategy (a) and the simultaneous strategy (b).

When routine coverage increases over time, the minimum threshold budget under the sequential strategy is only $40 million instead of $170 million. As annualbudget increases beyond this threshold value, the time to eradication of each disease decreases steadily, and for an annual budget of $200 million all diseases are eradicated by 2035 (Figure 8a). By comparison, under the simultaneous strategy, the minimum threshold budget is much larger ($110 million) and the time to eradication decreases only slightly as the annual SIA budget is increased (Figure 8b). The time to eradication remains very different under simultaneous versus sequential strategies under the scenario of rising routine vaccine coverage: for an annual budget of $150, million, Disease A, B, and C are eradicated by 2038, 2041 and 2047 respectively under the sequential strategy, but these events occur much later, at 2059, 2074 and 2083 respectively, under the simultaneous strategy. Hence, even when routine coverage is increasing over time, budget reallocation strategies such as the sequential strategy evaluated here can significantly accelerate eradication of all three infectious diseases compared to a simultaneous strategy. We note that these results do not include the cost of ramping up routine coverage, although accounting for this would not change our qualitative conclusions since the resulting costs would be the same for the sequential and simultaneous strategy, and routine immunization would probably continue after eradication. We also note that exploring whether to allocate funding to improving routine immunization or implementing more SIAs in real-world immunization programs would require a country-specific and disease-specific model.

### Case Importation Rate

Here we examine the effects of case importation on the time to eradication for the baseline sequential strategy with an annual SIA budget of $180 million. Case importation has two competing effects on the time to eradication. On one hand, moderate case importation can prevent local elimination of a disease: an ongoing epidemic in one population can "re-seed" other populations where the disease might otherwise have disappeared due to low prevalence, and thus delay elimination in those populations. On the other hand, sufficiently large rates of case importation could also synchronize epidemics in connected populations. As a result, all populations would experience an epidemic trough at the same time, meaning that "re-seeding" effects from other populations would not prevent local elimination in a given population. Because all three populations experience epidemic troughs at the same time and re-seeding is not possible, the chances of eliminating the infection in all three populations are higher in this scenario [34].

**Figure 9:**
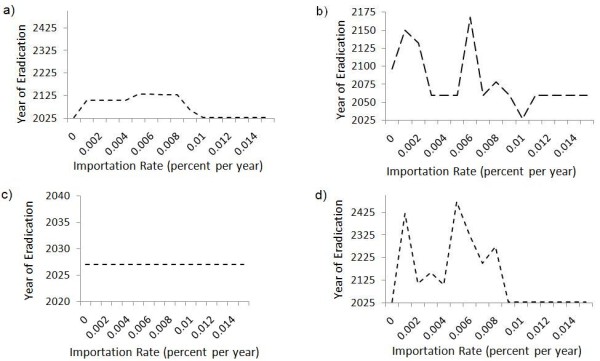
**Impact of case importation rate**. Year of eradication of all three diseases versus case importation rate, under the sequential strategy. In subpanel (a), importation rates are varied uniformly across all countries. In subpanel (b) case importation rate into and out of India varies while the migration rates between Nigeria and Afghanistan remain at the baseline value of 0.01% per year; Subpanels (c) and (d) show the same for Nigeria and Afghanistan respectively.

Because of these two competing effects, the impact of case importation on time to eradication in our model is complex. If the case importation rate between the three countries is higher than our baseline value (*m *> 0.0001/year), we observe that all three diseases are eradicated by 2025 (Figure 9a). Synchronization may contribute to this effect and is apparent for some of the diseases in Figure 1. However, for smaller values of the case importation rate (*m *< 0.0001/year) the time to eradication increases significantly and eradication tends to be delayed until 2100 or 2125. When *m *= 0, there is no case importation, and hence there can be no rescue effect due to re-seeding, making it easier for eradication to occur, hence, the time to eradication fall back to 2025 when *m *= 0 (Figure 9a).

The patterns are even more complex when case importation is varied for one country at a time. For example, when the India-Afghanistan and India-Nigeria case importation rates are changed while the Afghanistan-Nigeria case importation rates are held constant at baseline values, the year of eradication is highly variable across the range of case importation values explored (Figure 9b). This variability in year of eradication is even higher across a range of case importation rates into and out of Afghanistan (Figure 9d), although the same variability is not observed across a range of case importation rates into and out of Nigeria (Figure 9c).

**Figure 10:**
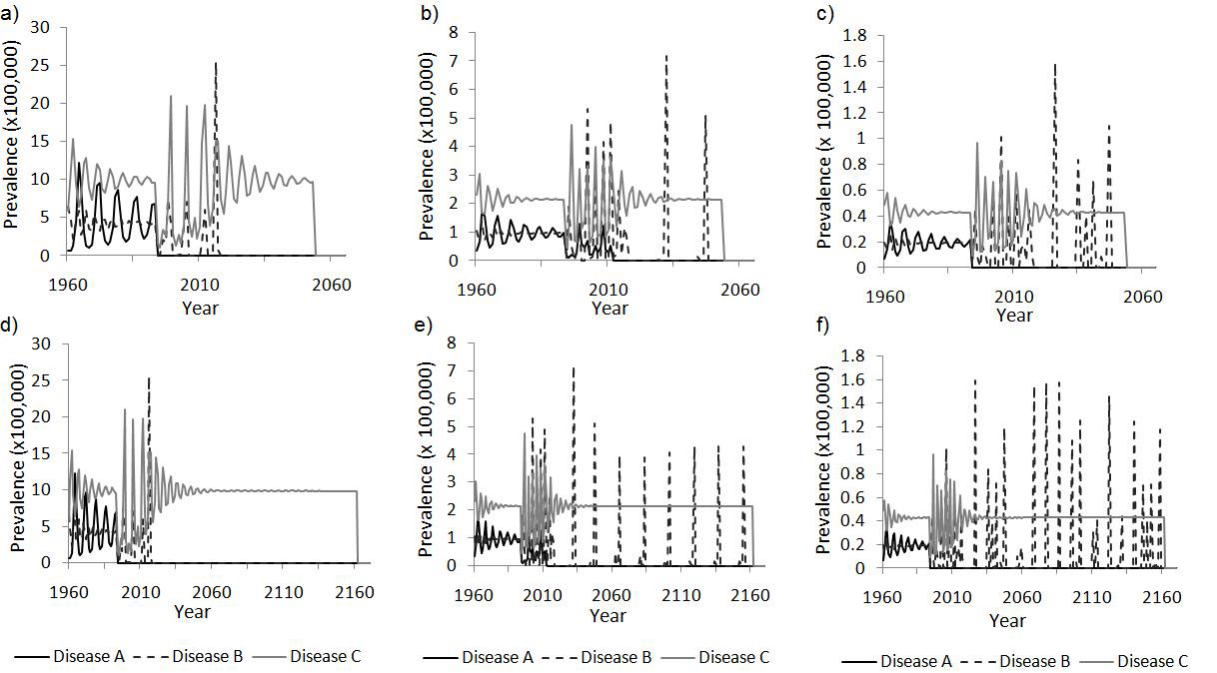
**Time series for similar case importation rates**. Prevalence of the three diseases over time in the sequential strategy, given a case importation rate of 0.05% per year into and out of India, for India (a), Nigeria (b) and Afghanistan (c), and given a case importation rate into and out of India is 0.06% per year for India (d), Nigeria (e) and Afghanistan (f). These two scenarios correspond to that of Figure 9b, where other case importation rates are held constant at baseline value of 0.01% per year.

To understand how small changes in case importation rate can lead to large changes in the time to eradication, we contrast the scenarios where case importation rates into and out of India occur at 0.05% per year versus 0.06% per year while other case import rates are held constant at baseline values (Figure 9b). In Figure 9b we observed that eradication of all three diseases occurs by 2060 at a rate of 0.05% per year, but it does not occur until 2168 at a rate of 0.06% per year. In a plot of disease prevalence over time corresponding to these two scenarios (Figure 10), we observe that the dynamics of Disease B are driving these contrasting outcomes. When case importation occurs at a rate of 0.05% per year, outbreaks of Disease B in Nigeria and Afghanistan are highly episodic and appear to be subject to local extinction (Figure 10a-c). Hence, eradication occurs by 2060. In comparison, when case importation occurs at 0.06% per year, outbreaks of Disease B become more regular and less episodic due to rescue effects, such that whenever prevalence is low in Nigeria, prevalence is often high in Afghanistan and vice versa (Figure 10d-f). As a result, all three diseases are not eradicated until 2168.

## Discussion

Here we analyzed an infectious disease transmission model where SIA programs for three hypothetical infectious diseases (A, B, C) in Nigeria, Afghanistan and India share a common budget. A global decision-maker can allocate differing amounts of money each year out of the common annual budget to SIA programs for the three diseases, and the budget determines vaccine coverage for each program. We compared a "simultaneous strategy" of dividing the budget equally among SIA programs each year for the three diseases to a "sequential strategy" where all annual SIA budget is first allocated to Disease A until Disease A is eradicated, at which point all annual SIA budget is redirected to Disease B until it is eradicated, and then finally to Disease C until it is eradicated.

We found a broad range of parameter values for which, under the same annual SIA budget, all three diseases could be eradicated relatively quickly (by 2030) under the sequential strategy, but none of them could be eradicated under the simultaneous strategy. Under the simultaneous strategy, the only way to eradicate all three diseases on a timescale as rapid as the sequential strategy was to triple the annual SIA budget--a considerable increase in expenditure. Large differences in time to eradication under the two strategies persisted for a range of scenarios, including scenarios of both constant routine coverage and rising routine coverage. We also found that, for certain values of the total annual budget close to the optimal values that minimize total long-term spending, the order of eradication can make a significant difference: under the sequential strategy, eradication of all three diseases happens much more quickly when Disease B is the first or second disease targeted--if Disease B is targeted last, eradication of all three diseases does not occur until at least 2177. For certain values of the budget close to the optimal budget we found that an interruption of SIA funding of several years close to the cusp of eradication can lengthen the time to eradication by many decades: this outcome can be summed up as "one step back, twenty more steps back". These results illustrate how budget allocation strategies can interact with the inherently nonlinear dynamics of disease transmission and herd immunity effects to cause surprisingly divergent outcomes under moderately different budget allocation strategies. Finally, we found that the predicted time to eradication was very sensitive to levels of case importation between the three countries. We emphasize that our model was simplified in order to illustrate certain principles around the interaction between budget prioritization and infectious disease dynamics, and is not intended for direct use in policy considerations. For policy considerations, it would be necessary to develop a country-specific and disease-specific approach.

Our objective was to illustrate how the nonlinearities of disease transmission (and herd immunity in particular) can amplify relatively modest changes in near-term budget allocations, resulting in enormous long-term differences in disease prevalence. Our objective was not to provide a tool that could be used to inform specific policies. This would require greater attention to factors such as transmission heterogeneity and seasonality; a broader spectrum of costs including opportunity costs and costs of treating infections and controlling outbreaks; a broader spectrum of health outcomes including use of disability-adjusted-life-years (DALYs) to allow sensible comparison between different strategies and different diseases in terms of total disease burden; the fact that cancelling SIAs for a given disease may not always be possible or desirable due to use of combined vaccines or joint administration of control programs; judicious use of discounting to weigh tradeoffs between short-term versus long-term outcomes in different birth cohorts; possible ethical concerns related to the increased incidence of all other diseases in the short term while funding goes specifically to the eradication one disease alone; higher vaccine unit costs required to reach the last remaining reservoirs of transmission (populations) that tend to be harder to reach; cost savings in simultaneous strategies due to being able to administer multiple vaccines in the same SIA; and incorporation of "hard restrictions" on how international donors support SIA programs. Another limitation of our model is that it is not age-structured with respect to transmission or vaccination target groups. Including age structure could impact the required threshold for eradication, or inform eradication strategies such as optimal target age groups for SIAs [35]. Future studies incorporating age-structure could therefore refine our results. However, we suggest that many of the nonlinear effects such as herd immunity will be conserved under more sophisticated treatments of this problem, which would thus exhibit similar amplification effects as observed here.

More sophisticated models could also be used to compare control measures for diseases with different modalities of transmission, such as measles versus malaria versus human immunodeficiency virus (HIV). Our model only applies to diseases that are transmitted through direct contact, i.e., person to person. Its results may not apply to diseases that can be transmitted through indirect contact (such as through infected food or water). The model also assumes homogeneous mixing, which does not apply at the country level. As a result, the predicted dynamics could differ from those of a model where a country is broken down into mixing subpopulations. Many of these issues could be addressed in future work, and a possible application of more sophisticated versions of this type of model is optimizing the portfolios of large international donors.

## Conclusions

We found that re-allocating budget among SIA programs for three hypothetical infectious paediatric diseases under a sequential eradication strategy can significantly speed eradication of all three diseases for the same annual SIA budget, under a range of model assumptions. Moreover, relatively modest differences in budget allocation in the near-term can result in very significant long-term differences in outcomes-- particularly the time to eradication--as a result of the amplifying effects of herd immunity and the nonlinearities of disease transmission. More sophisticated versions of such models may be useful to large international donors or other organizations as a planning tool or a tool for portfolio optimization, where choices must be made regarding how much funding to dedicate to different infectious disease eradication efforts.

## Abbreviations

SIA: supplementary immunization activities; SIRV: Susceptible-Infectious-Recovered-Vaccinated.

## Competing interests

The authors declare that they have no competing interests.

## Authors' contributions

CTB conceived of the study, contributed to the development of the research question, participated in its design, contributed to the development of the model and MATLAB code, and revised the manuscript. TF contributed to the development of the research question, developed the model code, carried out simulations and analysis, and drafted the manuscript. All authors read and approved the final manuscript.

## Pre-publication history

The pre-publication history for this paper can be accessed here:

http://www.biomedcentral.com/1471-2458/11/739/prepub
